# From hypereosinophilia to hypereosinophilic syndrome: real-world application of a two-tailed approach for HES diagnosis

**DOI:** 10.3389/fimmu.2025.1735131

**Published:** 2026-01-06

**Authors:** Stefania Nicola, Richard Borrelli, Irene Ridolfi, Luca Lo Sardo, Simone Negrini, Monica Fornero, Nicolò Rashidy, Federica Corradi, Iuliana Badiu, Eleonora Cerutti, Giulia Costanzo, Stefano Del Giacco, Giovanni Rolla, Luisa Brussino

**Affiliations:** 1 Struttura Complessa a Direzione Universitaria (SCDU) Immunologia e Allergologia, Azienda Ospedaliera (AO) Ordine Mauriziano di Torino – C.so Re Umberto, Torino, Italy; 2 Dipartimento di Scienze Mediche – Università degli Studi di Torino – C.so Dogliotti, Torino, Italy; 3 Struttura Complessa (SC) Farmacia Ospedaliera - Azienda Ospedaliera (AO) Ordine Mauriziano di Torino – C.so Re Umberto, Torino, Italy; 4 Medical Science and Public Health, University of Cagliari - Via Università, Cagliari, Italy

**Keywords:** hypereosinophilia, hypereosinophilic syndrome (HES), mepolizumab, two-tailed approach, heart involvement, EGPA, single-center report, EGID

## Abstract

**Background:**

Hypereosinophilic Syndrome (HES) is a rare disorder with a heterogeneous clinical presentation. If not recognized, it can lead to diagnostic delay and worse prognosis. Our study aimed to describe the real-world scenario of patients presenting with hypereosinophilia (HE), diagnosed with HES in an Italian Immunology Excellence University Centre. In addition, we also assessed the feasibility of a two-tailed approach for HES diagnosis, which consists of proceeding from the beginning with the differential diagnosis and systematic evaluation of organ damage.

**Methods:**

A retrospective observational single-center study was conducted. All patients underwent blood and instrumental tests to simultaneously identify HES etiology and any organ damage, through a process we called the “two-tailed approach”.

**Results:**

Two hundred forty-seven patients with HE referred to our center underwent the two-tailed approach. Due to either the presence of a straightforward underlying disease associated with HE, or the lack of sustained hypereosinophilia, 168 patients (68.0%) were excluded from the study. Seventy-nine patients (31 females, 39.2%) with a mean age of 54.9 years were finally diagnosed with HES. 19 (24.1%) patients were diagnosed with reactive HES, 15 (19.0%) with overlap HES, 1 (1.3%) with myeloid-HES, 10 (12.7%) with lymphocytic HES, and 8 (10.1%) with idiopathic HES. Sixty-three patients showed involvement of at least two organs: the lung (32/63, 50.7%), the skin (24/63, 38.1%), the bowel (23/63, 36.5%), and the peripheral nervous system (25.4%). Eight patients (8/63, 12.7%) showed heart involvement. The diagnosis was achieved in 4 ± 1.8 months, and no deaths were observed.

**Conclusion:**

HE is a common reason for consultations with allergists and clinical immunologists, and the two-tailed approach, which tests simultaneously for diagnosis and organ damage, should be implemented from the initial evaluation of patients with HE. The lower rate of idiopathic HES diagnosis and the higher frequency of heart involvement we found confirm the usefulness of the tool in reducing the risk of mistakes in classifying HES subtypes and the diagnostic delay, thus allowing prompt and tailored treatment and better outcomes.

## Introduction

Eosinophilia, defined as an absolute eosinophil count (AEC) > 0.5  × 10^9^/L, is a rather common condition, with a 1%–2% prevalence in the general population (1). On the other hand, a finding of an AEC >1500 cell/mcl, or hypereosinophilia (HE), is rare, with an estimated incidence of 6.3/100,000/year in the United States and Europe (2).

Indeed, eosinophilia and hypereosinophilia frequently require allergy or immunology consultations due to their association with organ damage or as an incidental finding (2).

In most cases, the clinical manifestations associated with HE clearly depict the underlying diseases, so diagnostic bickering fades quickly. For example, the presence of HE with cytopenia or lymph node enlargement immediately raises the suspicion of an underlying hematological disease, and the patient is immediately referred to the proper specialist. On the other hand, a patient with HE, asthma with aspergillus sensitization, lung infiltrates, and an increase in total IgE is highly suggestive of allergic bronchopulmonary aspergillosis (ABPA). Finally, one with HE, severe asthma, chronic rhinosinusitis with nasal polyps, ground glass lung involvement, skin purpura, and multiplex mononeuritis is likely to be affected with eosinophilic granulomatosis with polyangiitis (EGPA). **Table 1** shows the most common conditions and diseases that should be ruled out before considering a diagnosis of HES (non-exhaustive list).

**Table 1:** The most common conditions and diseases that should be ruled out before considering a diagnosis of HES (non-exhaustive list).

Cause or diagnosis	Subgroups of disease and details
Myeloid neoplasms	Chronic eosinophilic leukemia (CEL), myeloproliferative neoplasms (MPN), acute myeloid leukemia
Lymphoid neoplasms	T-cell lymphomas, B-cell lymphomas
Myeloid/lymphoid neoplasms with gene rearrangements	PDGFRA, PDGFRB, FGFR1, PCM1-JAK2 rearrangements
Parasitic infections	Helminths (Strongyloides, Schistosoma, Toxocara, etc.), protozoa
Allergic diseases	Asthma, atopic dermatitis, allergic rhinitis
Drug reactions	Antibiotics (β-lactams), allopurinol, NSAIDs, others
Autoimmune/rheumatologic diseases	Eosinophilic granulomatosis with polyangiitis (EGPA), connective tissue disorders, vasculitis
Other infections	Fungal (Aspergillus), viral (HIV), bacterial, mycobacterium
Malignancies (non-hematologic)	Solid tumors (lung, GI, ovary, etc.)
Endocrine disorders	Adrenal insufficiency
Immunologic syndromes	IgG4-related disease, Hyper-IgE
Gastrointestinal diseases	Inflammatory Bowel Disease, celiac disease
Idiopathic hypereosinophilia/HES	No identifiable cause after exhaustive workup

In the case of no promptly identifiable systemic diseases, a thorough assessment of both the causes of HE and organ damage should be done. The presence of HE for more than four weeks, associated with end-organ damage and no other plausible causes of HE, is in fact conventionally defined as Hypereosinophilic Syndrome (HES) (3), a rare disorder (4) with a highly heterogeneous clinical presentation and a very bad prognosis that also includes exitus if not recognized or left untreated (5).

Even though the skin, lungs, and gastrointestinal tract are the most frequently involved, almost all organs can be a target of the effects of long-lasting sustained hypereosinophilia (6, 7). In addition, the eosinophilic involvement of many organs, such as the heart, can be completely asymptomatic for a long period and then suddenly manifest itself with a serious and intractable clinical picture (8).

Despite the fact that many attempts have been made to provide diagnostic and classification algorithms for HES (9–12), the diagnostic delay is still considerable, in some cases reported up to 20 years (13). In addition, due to the failure to share a rational approach to HES, many patients with HES are still misclassified as idiopathic HES.

Recently, many international consensus (12) focused on the need for a complete diagnostic workup based on a whole assessment of potential causes of HE and the systematic evaluation of organ damage starting from the beginning of the patient’s medical history. Nevertheless, the importance of a proper diagnosis and organ damage assessment has been known for many years (14, 15), even though to date, no data concerning the application of the algorithm in a real-world setting are available.

Hence, our study aimed to describe the real-world scenario of patients presenting with HE not associated with other diseases that were later diagnosed with HES in an Italian Immunology Excellence University Centre. Moreover, we also aimed at highlighting the feasibility of the aforementioned diagnostic assessment, which we defined as a “two-tailed approach” in improving the HES diagnosis and looking for early organ damage.

## Patients and methods

A retrospective observational single-center study was conducted. All the adult patients referred for hypereosinophilia (AEC > 1500 cells/mcl on two or more occasions) to the Advanced Unit of Immunology and Allergy of the A. O. Ordine Mauriziano, Turin, Italy, from 2010 to 2020, were included.

On the other hand, the lack of the release in the informed consent or the presence of an AEC < 1500 cell/mcl represented exclusion criteria. Moreover, all the patients referred with hypereosinophilia sustained by another clearly identified cause (i.e., EGPA, ABPA, IgG4-related disease, systemic mastocytosis, infections, inflammatory bowel disease, systemic vasculitis, immunodeficiency, and other autoimmune diseases) (16) were excluded from the analysis.

The study was approved by the local ethics committee (Comitato Etico Territoriale Interaziendale (CET) “A.O.U. Città della Salute e della Scienza di Torino”, protocol code 665.743—dated 10th November 2021), and completely conducted following the Declaration of Helsinki and the latest Best Clinical Practice guidelines. All the enrolled patients released their informed consent for publication.

### Data collection

For each patient, data concerning age, sex, smoking habits, clinical examination, a deep medical history including atopic comorbidity, travels, food habits, and home treatments were collected. All the data were retrospectively collected based on their electronic medical records.

### Two-tailed algorithm simultaneous approach

According to the 2007 recommendations (14) and based on the extensive diagnostic work-up later recommended in 2010 (15), starting from 2010 we decided to follow a strict medical diagnostic algorithm in all the patients referred to our immunology center for HE. By the first immunology outpatient visit, all the patients were prescribed the medical tests listed below, and the first follow-up visit was usually set between two and four months later.

Therefore, all the enrolled patients followed a two-tailed simultaneous approach to evaluate organ involvement and highlight the underlying cause for HE or HES.

Hence, due to the recommendations of 2007 and 2010, all the patients underwent the exams listed below as a standard-of-care assessment for a rare disease.

### Blood tests

All the patients, regardless of symptoms, underwent the following blood tests: complete blood count, liver and kidney function, ESR and CRP, urinalysis, protein electrophoresis, thyroid hormones, CPK, LDH, and blood urea nitrogen/creatinine (17).

### HES etiology

To exclude a *reactive HES (r-HES)*, all the patients were assessed for:

- *Allergic disorders*: complete medical history, including asthma and CRSwNP, was collected; skin prick tests or *in-vitro* tests (specific IgEs) for perennial and seasonal aeroallergens (grass, ragweed, mugwort, tree pollens, pet dander, house dust mites, and molds), and for anisakis were done in all the patients.
- *Drug Rash with Eosinophilia and Systemic Symptoms (DRESS):* routine home treatments (i.e., ASA, TMP/SMZ, penicillins, and beta-lactams) were recorded. In addition, anticonvulsants, sulfones and sulfonamides, and allopurinol intake were assessed.
- *Helminth infections:* stool parasite search, IgG and IgM antibody testing (Thermo Scientific) for Toxocara canis, Schistosomes spp., Trichinella spiralis, Strongyloides stercoralis, and Echinococcus were performed in all the patients. In addition, skin prick tests for anisakis were also performed.
- Other infections: Serology for HIV, HBV, HCV, and Interferon Gamma Release Assay were performed in all the patients.
- *Malignancy-associated HES*, including lymphoma (T-cell lymphomas and Hodgkin’s lymphoma) and solid tumors, that is, uterus, lung, stomach, colon, bladder, and pharynx, were ruled out through ultrasound and/or abdomen/chest CT scans. In the case of a patient with recent (<6 months) unremarkable imaging already done, no other radiological exams were performed.
- In addition, drug abuse, e-cig use, and the potential risk for metal inhalation were also assessed (10).

To exclude a *myeloid (m-HES) or lymphocytic (l-HES) HES (*14*)*, the following data were assessed:

- *Myeloid HES*: in addition to peripheral blood smear examination, the mutations in FGFR1, PDGFRβ, PDGFRα, PCM1-JAK2, and the fusion of the FIP1L1 and PDGFRA genes were assessed.
- *Lymphocytic HES*: to exclude the presence of a clone of eosinophil cytokine-producing T lymphocytes in all the patients, a lymphocyte subset (CD3+, CD3+CD4+, CD3+CD8+, CD3+CD4-CD8-, CD3+CD4+CD8+, CD3-CD56-, CD5+, CD19+, CD20+, CD38+CD27+IgM-, CD3+CD56+) and the TCR gene rearrangements were searched.

To exclude an *overlap form of HES*, the patients underwent the following evaluations:

- *Allergic Bronchopulmonary Aspergillosis* (ABPA): following the last medical algorithm (18) for ABPA diagnosis, all the patients with a history of asthma, cystic fibrosis, or chronic obstructive pulmonary disease (COPD) and chest CT scan abnormalities underwent allergy tests for Aspergillus fumigatus (Af) sensitization and total IgE. In case of positive results, minor criteria for ABPA and Af components were assessed.
- *Gleich’s syndrome* (cyclic angioedema with hypereosinophilia): in the case of a patient complaining of cyclic or recurrent angioedema and peripheral blood HE, with or without polyclonal IgM increase, surface marker expression was assessed on T cells by flow cytometry. An aberrant CD3-CD4+ T-cell population is commonly detected (19).
- *Eosinophilic granulomatosis with polyangiitis* (EGPA): a history of asthma and CRSwNP, associated with a necrotizing vasculitis process, are the main features for the suspicion of EGPA (20). In this case, the patients followed an EGPA complete diagnostic work-up, including an ear, nose, and throat (ENT) evaluation and Antineutrophil Cytoplasmic Antibodies (ANCA) lab test.
- *Eosinophilia Myalgia syndrome*, *IgG4-related disease, Sarcoidosis, autoimmune lymphoproliferative syndrome (ALPS), Omenn syndrome*, and *hyper-IgE syndrome* are quite rare clinical conditions. In the suspicion of these disorders, a specific workup was also followed (19).

In the case of HE and organ damage in which eosinophils play critical pathological roles, with some but not all criteria of an eosinophil-associated disorder being met, the patients were diagnosed with overlap HES.

*Idiopathic HES (i-HES):* after all primary and secondary HE causes have been excluded, the patients were diagnosed with I-HES (19).

### Early organ damage detection

Regardless of symptoms, all enrolled patients underwent the following investigations since the first outpatient consultation to highlight any early organ damage (8).

- *Lung involvement: A* chest X-ray was performed on all the patients, if not performed elsewhere in the last 6 months. In the case of any further suspicion of lung involvement, an HRCT chest scan and lung function tests with diffusion of carbon monoxide (DLCO) were also obtained. Bronchoscopy with bronchoalveolar lavage and lung biopsy were considered third-level exams after a complete pulmonologist work-up was done.
- *Heart involvement*: Due to the lack of clinical symptoms at the beginning of heart involvement (8), all our patients underwent blood tests to look for abnormalities in NT-pro-BNP and serum troponin T and I levels. A transthoracic echocardiogram and an electrocardiogram were also performed. In case of abnormalities, a cardiac MRI was performed.
- *Spleen and liver involvement:* all the patients underwent abdomen US to detect any liver or spleen enlargement or inhomogeneity.

The following consultations were also performed if the patient complained of any symptoms suggestive of organ damage.

- *Skin:* The patient complaining of pruritus, urticaria, angioedema, psoriasis-like signs, itchy papules, nodules, or mouth ulcers was referred to the dermatology consultant, who performed a skin biopsy.
- *GI tract:* In the case of upper or lower GI symptoms, including nausea, vomiting, diarrhea, weight loss, heartburn, dyspepsia, or abdominal pain, the patient underwent upper or lower endoscopy with biopsy, aiming to evaluate tissue eosinophilia.
- *Central or peripheral nervous system (CNS or PNS)*: if the patient showed any form of sensory or motor neuropathy, headache, visual abnormalities or visual loss, a history of myelitis, meningitis, amnesia, or recent stroke, then electromyography, electroneuronography, and brain MRI were done.
- *Hematological system:* in case of anemia, leukopenia, low platelets, or increased levels of vitamin B12 and LDH, the patient was referred to a hematologist, and a bone marrow biopsy was then performed. Moreover, the history of deep vein thrombosis or embolic manifestations was considered an indication for hematologic consultation.
- In the case of a patient complaining of other signs or symptoms not listed above, targeted medical tests were performed, to exclude any potential organ damage or comorbidity.

If only one organ was being affected by eosinophil-induced damage (e.g., chronic eosinophilic pneumonia, eosinophilic gastroenteritis…), the HES was classified as associated with single-organ involvement, or “eosinophil-associated single-organ disease” (2).

In patients with long-lasting, unexplained, and asymptomatic HE, who did not show any organ damage, a diagnosis of “hypereosinophilia of undetermined significance” (HEus) was made (21).

**Figure 1:**
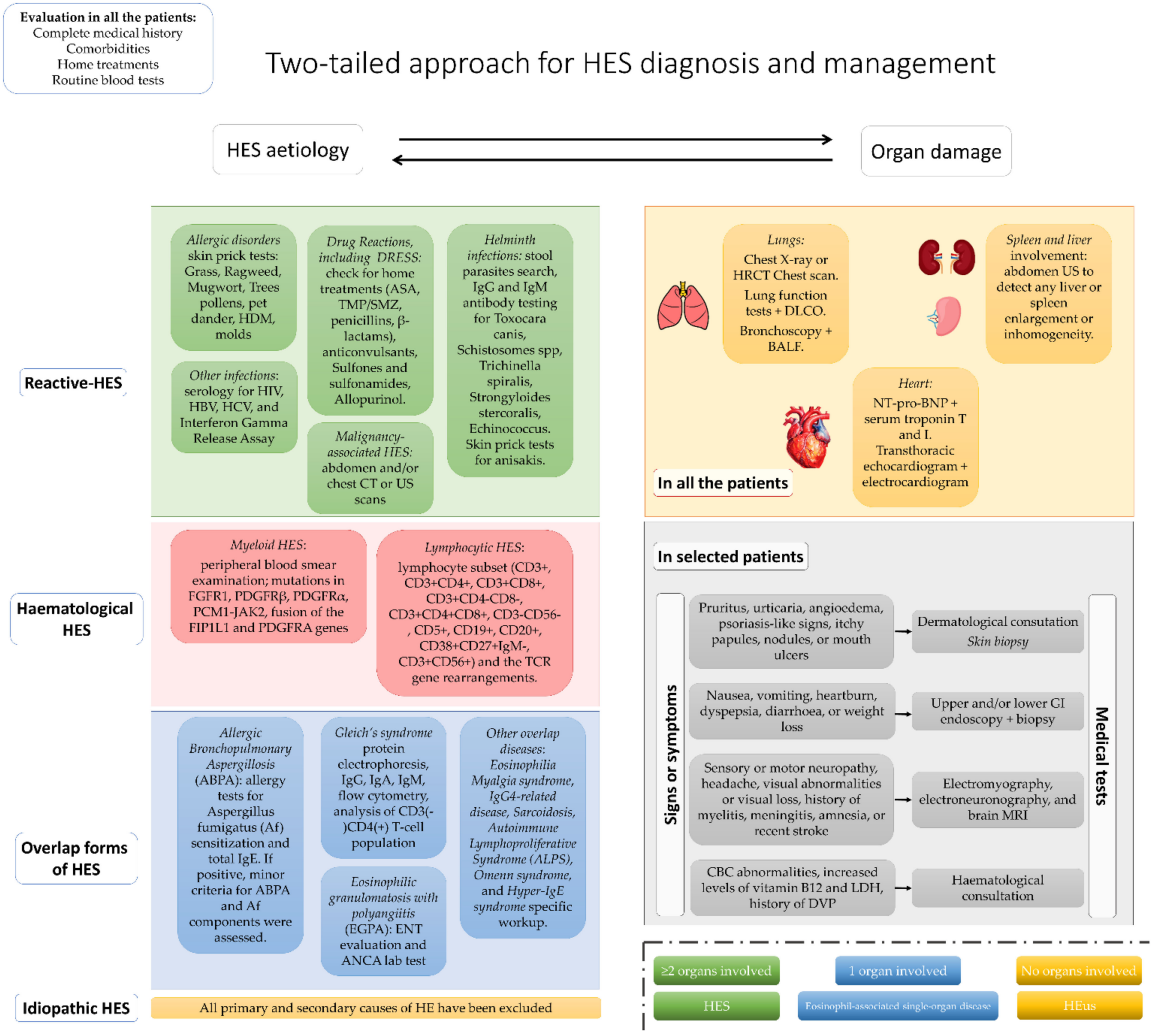
Two-tailed diagnostic approach used in the study.

**Figure 1** shows the diagnostic flowchart that we used in our study.

### Evaluation of diagnostic delay

Based on the electronic health record (EHR) of the enrolled patients, the time for the diagnosis was calculated as the time interval (months) between the first immunological examination at our center and the formulation of the diagnosis.

### Statistical analysis

Statistical analysis was performed using IBM SPSS Statistics for Windows software, version 28 (IBM Corp., Armonk, NY, USA). A descriptive analysis of the studied variables was conducted. The normality of the distribution of continuous variables was evaluated with the Shapiro-Wilk and the Kolmogorov-Smirnov tests. The distribution of the qualitative variables in absolute number and percentage has been reported, and the relative frequency tables have been created. For the quantitative variables, interquartile ranges were calculated based on the normality of the distribution, mean, standard deviation, or median. All the data were first analyzed with a Kolmogorov-Smirnov normality test. Data comparison was then performed using the Student T-test in the case of normally distributed data. Otherwise, the Mann-Whitney non-parametric test for independent samples was used. The comparison between different HES groups was done with a Kruskal-Wallis independent test, whereas the comparison between single HES groups was done through a Mann-Whitney test.

## Results

**Figure 2:**
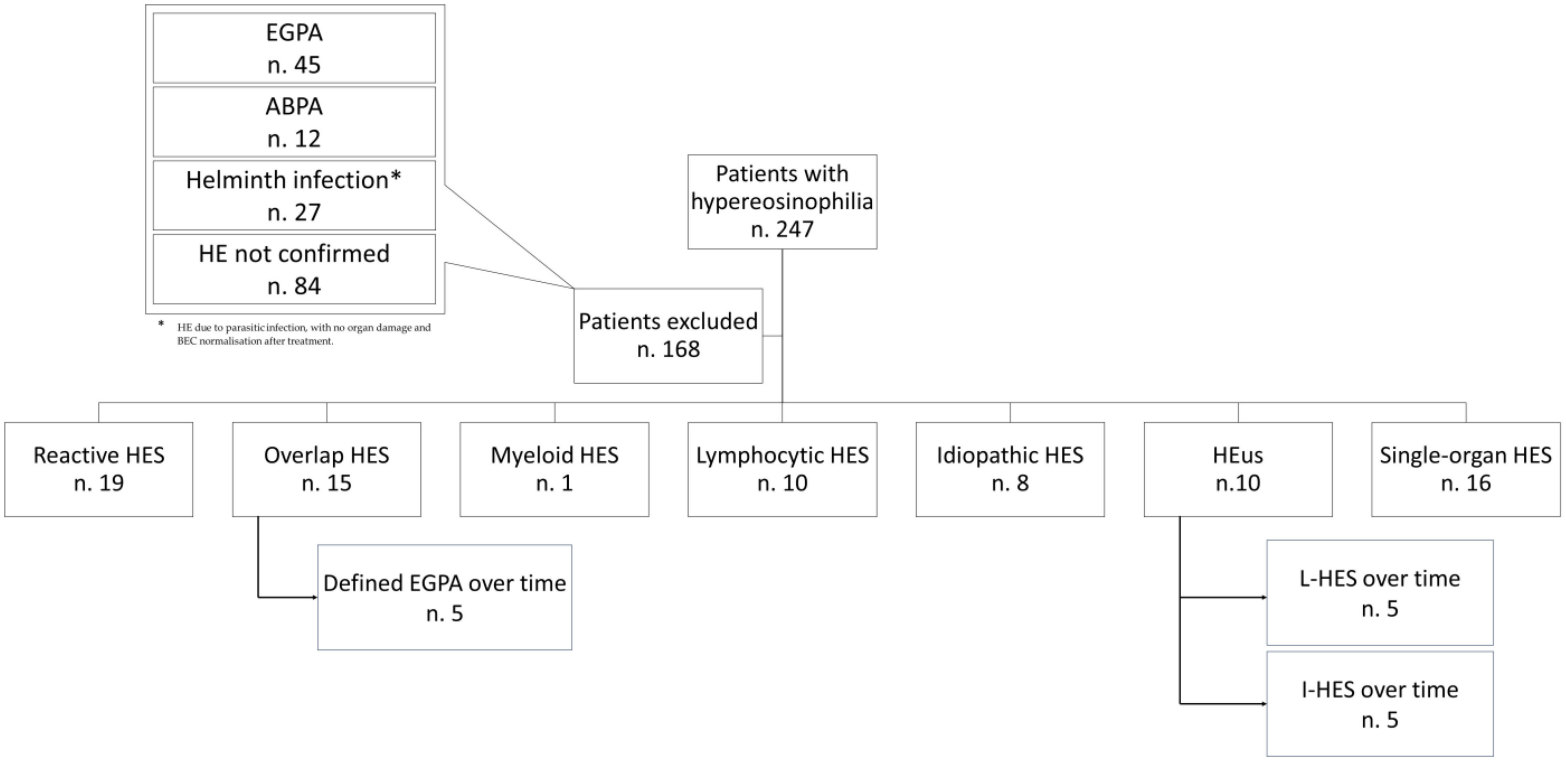
Study design and methods flow-chart.

A total of 247 patients with hypereosinophilia were enrolled. Due to either the presence of a straightforward underlying disease associated with HE or the lack of sustained hypereosinophilia, 168 patients (68.0%) were excluded from the study and not considered in the statistical analysis, as shown in **Figure 2**. Briefly, 45 patients were diagnosed with EGPA and 12 with ABPA. Twenty-seven patients had HE due to parasitic infection, with no organ damage and AEC normalization after treatment. Finally, in 84 patients, the levels of eosinophils were not sustained over time.

Therefore, 79 patients (31 females, 39.2%) with a mean age of 54.9 years (range, 14–83 years) were included in the study. The mean AEC at the diagnosis was 5328.2 ± 1032.3 cells/mcl. The median total IgE value was 1670 ± 1676 kU/L, and 21 patients were atopic (26%).

### HES distribution using the two-tailed algorithm

**Figure 3:**
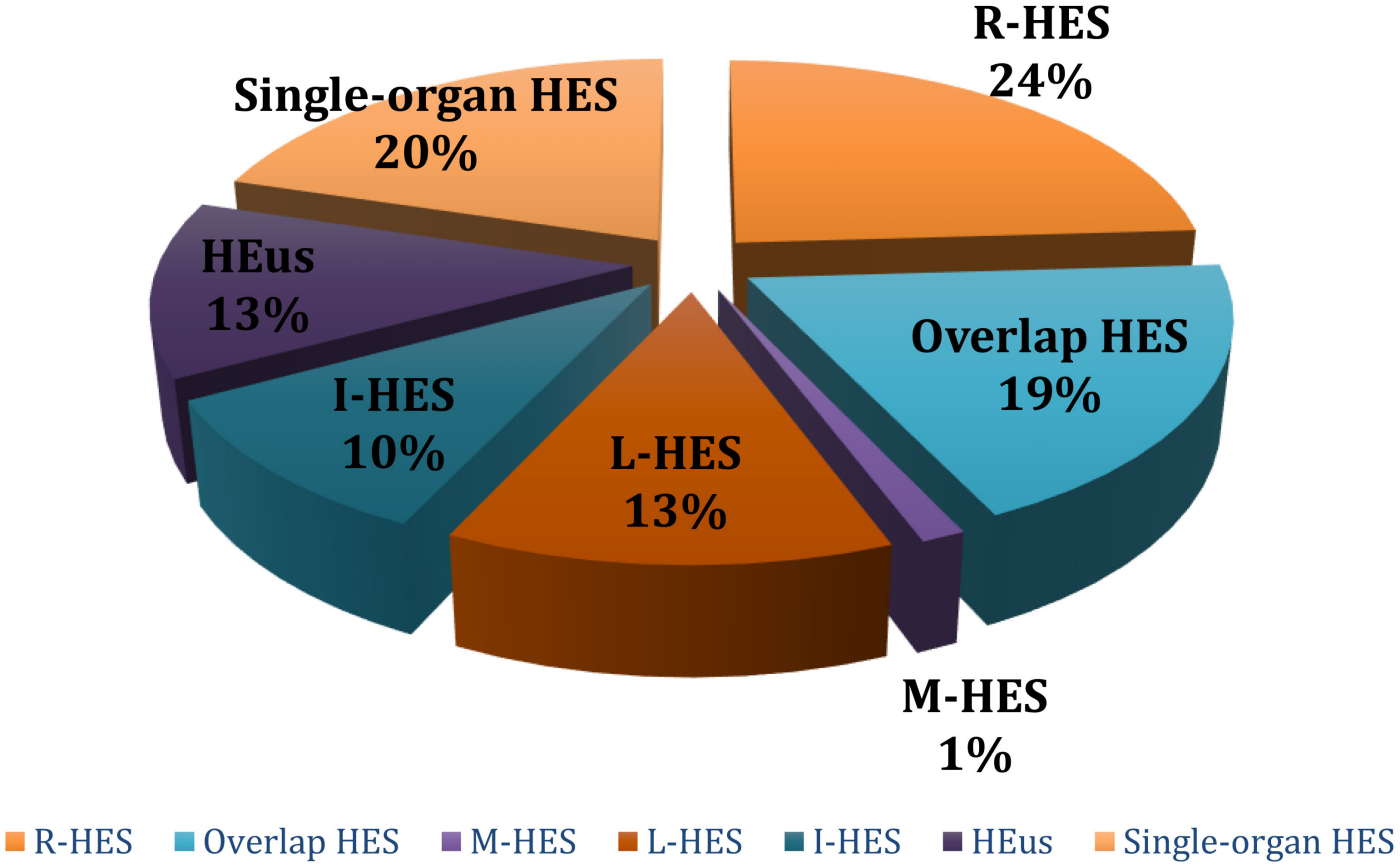
HES distribution using the two-tailed algorithm.

At the end of the diagnostic process, the patients were classified as follows: 19 (24.1%) patients were diagnosed with Reactive HES, 15 (19.0%) with Overlap HES, 1 (1.3%) with Myeloid-HES, 10 (12.7%) with Lymphocytic HES, 8 (10.1%) with Idiopathic HES, and 10 (12.7%) with HEus. In addition, 16 patients (20.3%) received a diagnosis of single-organ HES, as shown in **Figure 3**. **Supplementary Table S1** summarizes the different etiologies of HES, the demographic data, clinical manifestations, treatments, and laboratory values.

### Reactive HES - HES-R

Nineteen patients (19/79, 24%) were diagnosed with an HES-R, as shown in **Table 2**. Five patients (5/19, 26.3%) suffered from severe type 2 asthma associated with CRSwNP, 9 had confirmed helminthiasis (47.4%), 4 showed drug reactions with systemic symptoms (21.0%), and in just one case (5.3%) HE was associated with a malignant sarcoma.

**Table 2:** Distribution of variables in the different R-HES subtypes.

R-HES subtypes	Patients, *n* (%)	Age, yrs (range)	Females, *n* (%)	EOS, cell/mcl (mean ± SD)	Total IgE, KU/L (mean ± SD)
Asthma and CRSwNP	5 (26.3%)	50.4 (29–71)	2/6 (33.3%)	2180.0 ± 476.5	878.6 ± 762.4
Helminths	9 (47.4%)	57.5 (43–71)	4/6 (66.7%)	3410.0 ± 2756.9	2106.8 ± 1016.2
Drug reactions	4 (21.0%)	47.4 (27–67)	–	2968.0 ± 832.5	60.5 ± 35.0
Malignancy	1 (5.3%)	73	–	3160.0	156

Among patients diagnosed with a helminth infection, two had positive Strongyloides stercoralis IgG antibodies, one positive Toxocara canis IgG antibodies, and one patient showed a positive skin prick test for anisakis. Following the 2010 recommendation of hypereosinophilia (15), all the patients were treated with antihelminths (albendazole 400 mg once a day for a three-day course), with clinical improvement and AEC reduction.

In patients with DRESS, the culprit drugs were carbamazepine in one patient, cefixime in one patient, and allopurinol in two cases.

A patient was diagnosed with Gleich’s syndrome, but due to the clonal pattern of his T-lymphocyte subset, we decided to include it among L-HES.

In the whole R-HES group, the mean BEC was 3257.3 ± 2363.2 cells/mcl, and the mean level of serum total IgE was 1536.3 ± 1408.57 KU/L. The mean onset age was 56.15 years (range 27-73), and 6 (31.6%) were females. The distribution of variables in the different R-HES subtypes is shown in **Table 1**.

### Myeloid and lymphocytic HES - M-HES and L-HES

A patient (male, 46 years old) was diagnosed with M-HES. He showed moderate hypereosinophilia (2450 cells/mcl) and a marked increase in vitamin B12 (>3000 pg/ml). The bone marrow biopsy confirmed the suspicion of myeloproliferative syndrome, and FIP1L1-PDGFRA fusion was found.

In ten patients (10/79, 12.7%), a lymphocytic variant of HES was diagnosed. BEC at diagnosis was highly variable in these patients, with a mean AEC of 4619 cells/mm^3^ (range 1770–10410 cell/mcl).

All patients showed abnormalities in the TCRγ pattern, that is, monoclonal in one case and oligoclonal in the remaining ones (data not shown).

### Overlap form of HES

The Overlap HES diagnosis was made during the first months of follow-up when the two-tailed approach was used. At that time, the clinical distinction between HES and other well-defined diseases, mainly EGPA (21) was unclear, and the patients were then classified as affected with an overlap form of HES/EGPA.

According to the diagnostic and classification criteria (21, 22), and based on their clinical presentations, fifteen patients (15/79, 19.0%) were diagnosed with an overlap form of HES/EGPA.

Among them, six patients were female (6/15, 40%) and globally had a mean AEC of 12162.0 (± 9943 cell/mcl). At the disease onset, none of them showed ANCA positivity. They all had a clinical history of asthma with or without nasal polyps. Five patients (33.6%) also had skin involvement (4 palpable purpura, one urticaria), eight (53.3%) showed peripheral nervous system involvement (4 polyneuropathy, four multiplex mononeuritis), two (13.3%) had glomerulonephritis, and two (13.3%) had gastrointestinal involvement. In five patients (33.3%), the heart was also affected: 3 of them showed myocarditis, and two complained of symptoms suggestive of coronary artery vasculitis. Nine patients (60%) also performed a biopsy, according to the suspected organ damage: five patients underwent a skin biopsy, two a kidney sample, and two a colon biopsy. No vasculitis was demonstrated in all the biopsies, while all the samples showed a marked eosinophilic infiltrate. The histological exam was not performed in six patients, mainly due to the ongoing immunosuppressive treatment. During the ten-year follow-up, 5/15 (33.3.%) patients became positive for p-ANCA and, based on their clinical picture, together with the autoantibody pattern, were then classified as EGPA and not considered for further statistical analysis.

### Hypereosinophilia with unknown significance

Ten patients (12.7%) showed persistent hypereosinophilia (AEC 3123 ± 1542.8 cells/mcl) with no evidence of organ damage or other known cause of HE.

The mean age at onset was 54 years (range 16-74), and the sex distribution slightly favored the male sex (6/10, 60%).

Seven patients (70%) developed an idiopathic HES at the end of the ten-year follow-up period, whereas three (30%) showed hematologic involvement with lymphocyte abnormalities.

### Idiopathic HES

Eight patients (8/79, 10.1%) were diagnosed with idiopathic HES, with a mean peripheral eosinophilia of 4968 ± 1433 cells/mcl). The mean age at onset was 65.37 years (range 29–83), and 5 (62.5%) were females.

None showed positive skin prick tests nor had a medical history suggestive of allergic diseases. Any reactive, myeloid, or lymphocytic HES causes were excluded.

### Comparison of peripheral absolute eosinophil counts among different HES groups

Peripheral eosinophilia was higher in patients with overlap HES than in the other patient groups, as shown in **Figure 2**. The comparison between Overlap HES and the other HES groups showed significantly higher levels of AEC in the first group (*p* < 0.001).

### Organ involvement

Sixty-three patients (63/79, 79.8%) showed involvement of at least two organs.

The most frequently involved organ was the lung, which was affected in 32 patients (32/63, 50.7%), followed by the skin (24/63, 38.1%), the bowel (23/63, 36.5%) and the peripheral nervous system (16/63, 25.4%). Heart involvement was found in 8 (8/63, 12.7%) of our patients during the follow-up. Kidney involvement (4/63, 6%), deep venous thrombosis (2/63, 3%) and central nervous system involvement (1/63, 1.5%) were far less commonly observed in our cohort, as shown in **Figure 3**.

### Single-organ HES

Sixteen patients (16/79, 20.2%) were diagnosed with single-organ HES: seven patients had gastrointestinal involvement, eight had pulmonary involvement (chronic eosinophilic pneumonia), and one had eosinophilic fasciitis. The mean age at onset was 44.9 yrs (range 17-71), and 7 were females (46), with a mean AEC of 4118 ± 2222 cell/mcl.

### Correlations between AEC and organ damage

**Figure 4:**
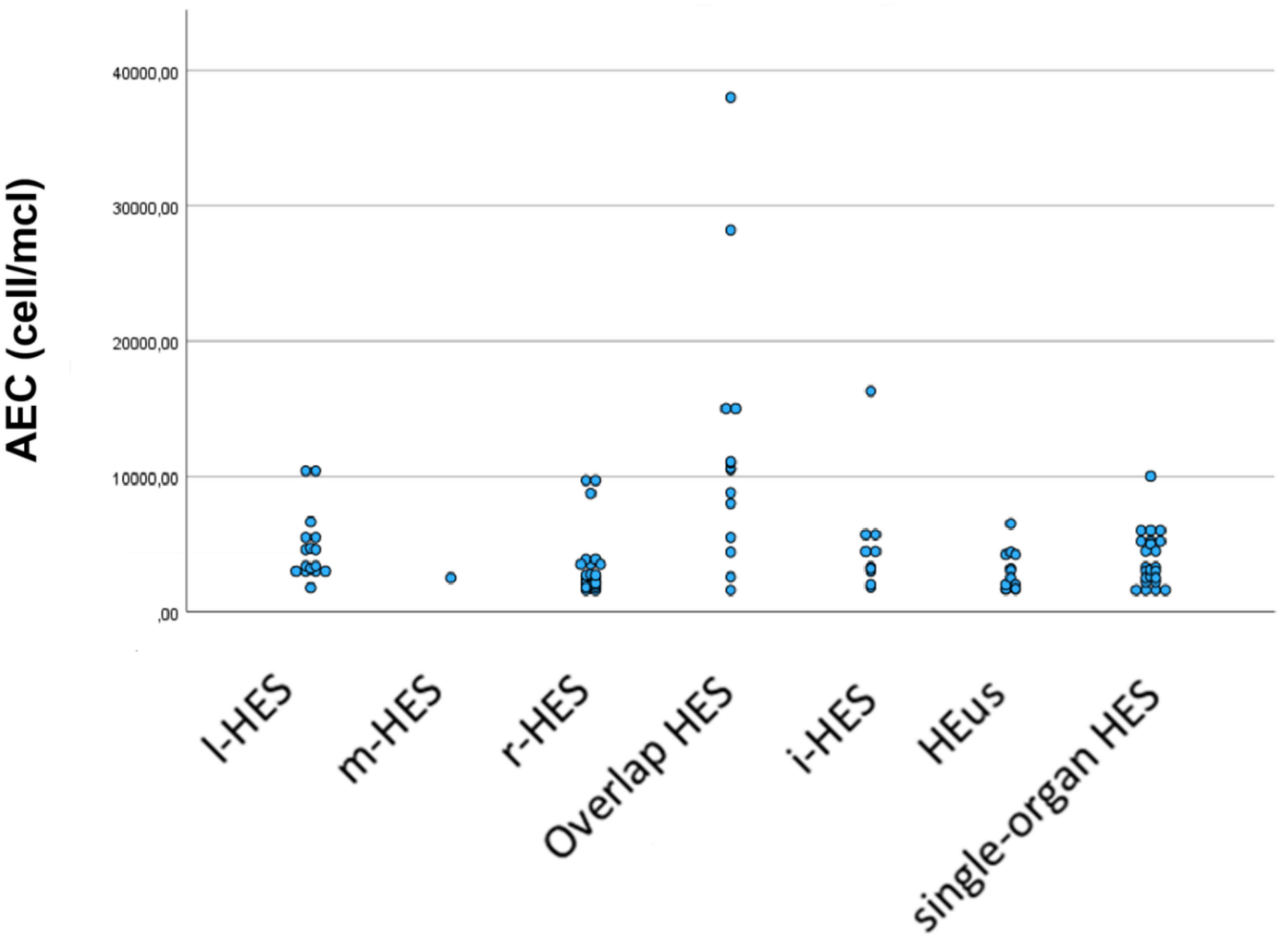
Comparison of eosinophilic levels among different HES groups.

**Figure 5:**
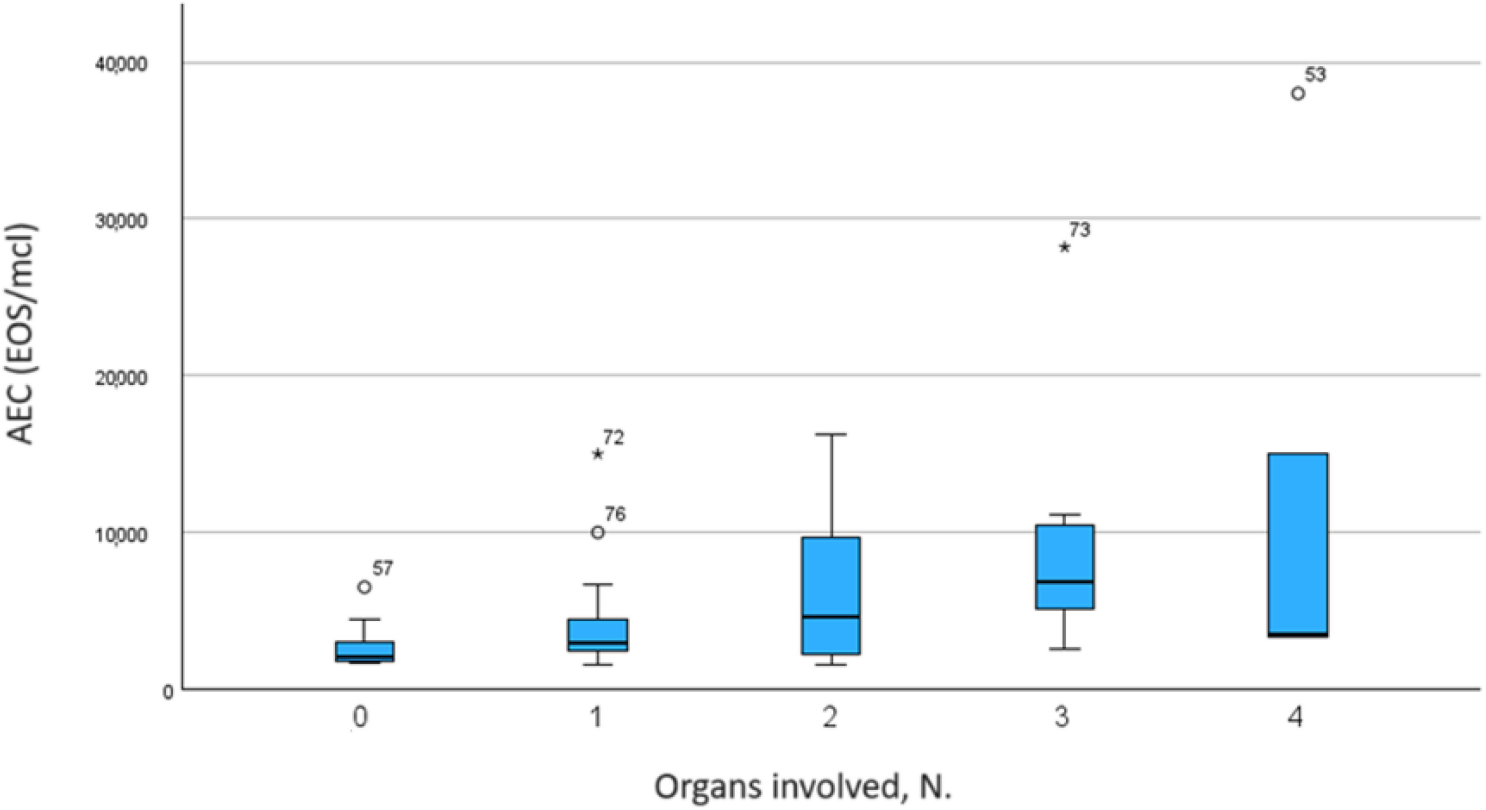
Correlations between AEC and number of involved organs.

In the enrolled cohort, the AEC positively correlated with the number of involved organs, as shown in **Figures 4**, **5** (*ρ* = 0.216, *p* = 0.027).

**Figure 6:**
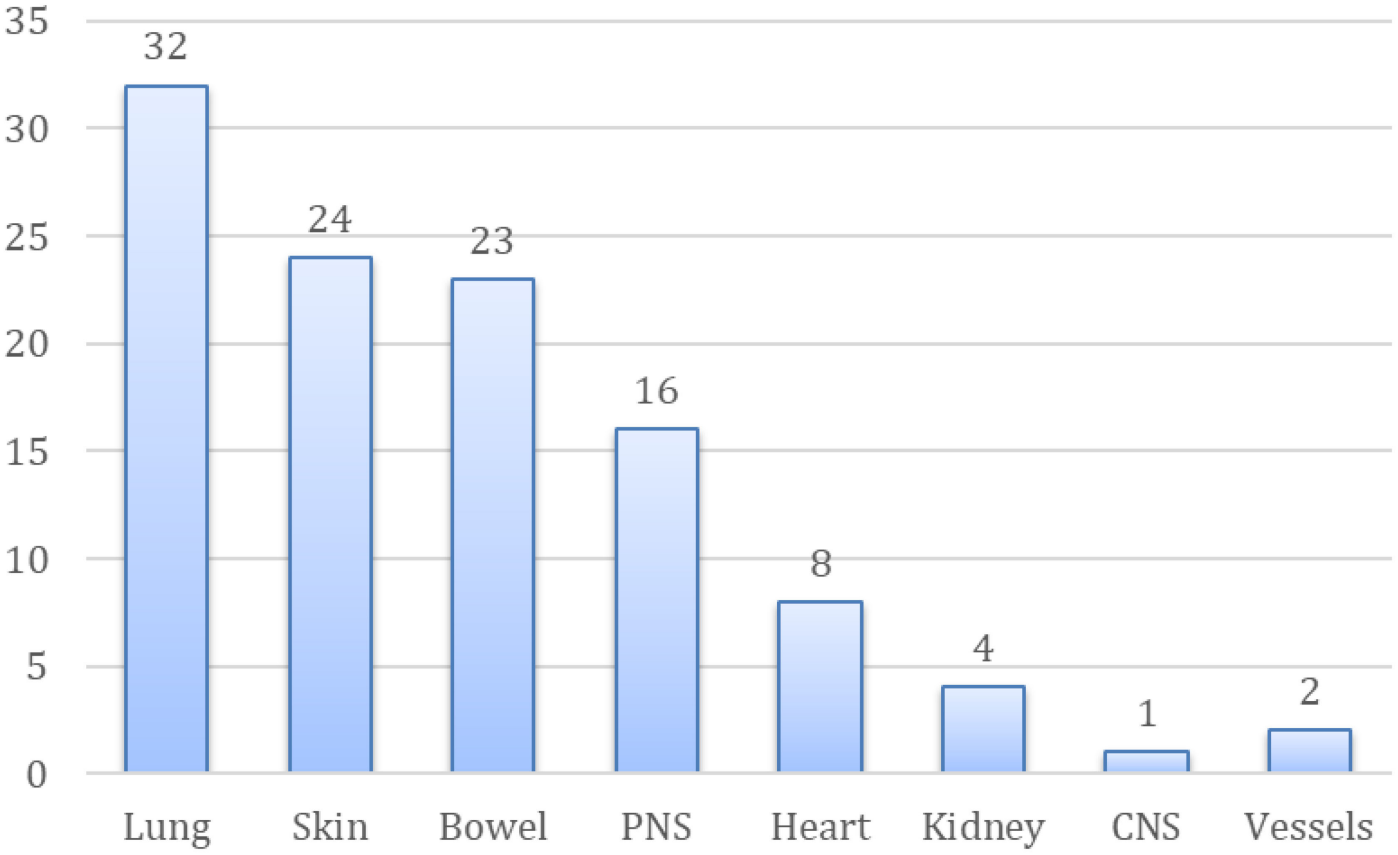
Distribution of organ involvement (number) in the enrolled cohort.

**Figure 7:**
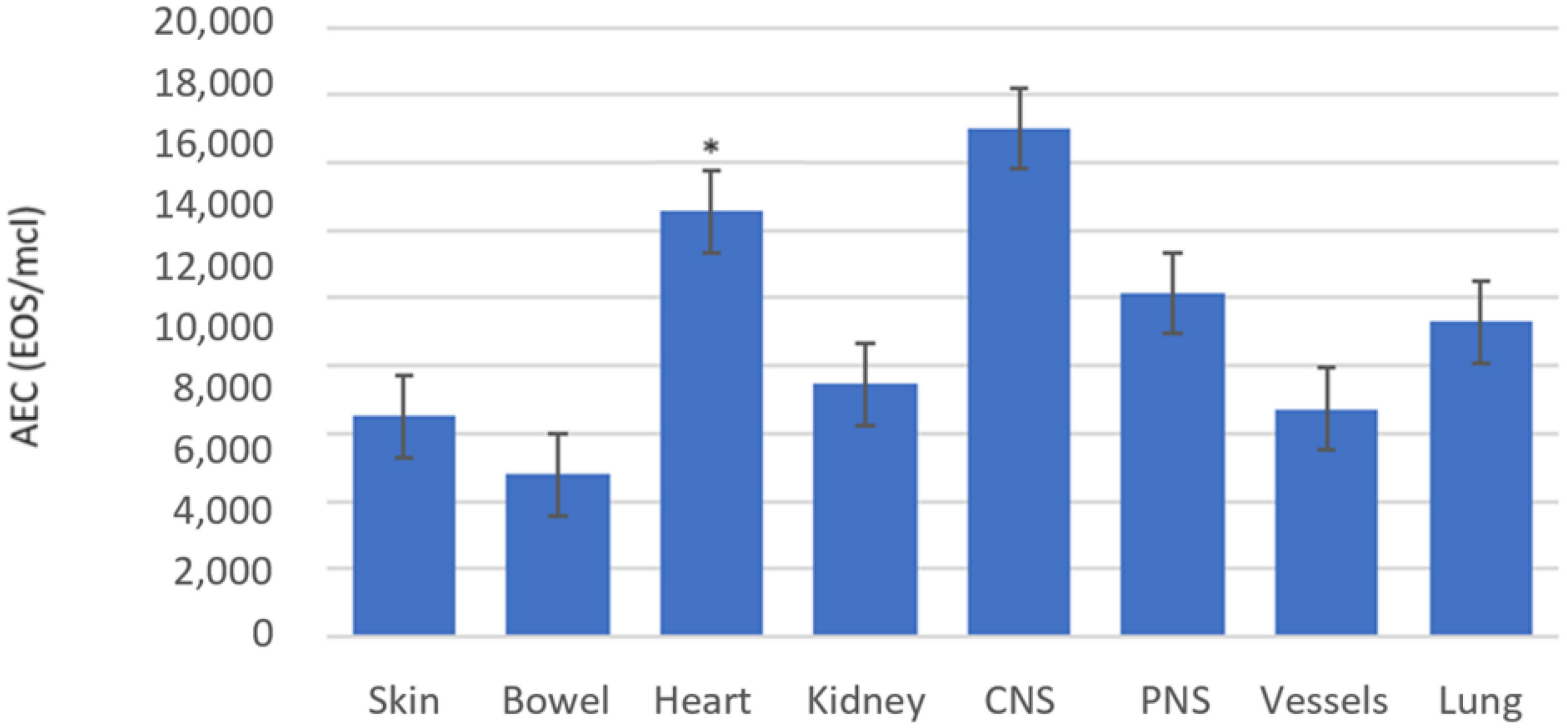
Mean AEC according to organ damage.

In addition, peripheral blood hypereosinophilia was significantly higher in the groups of patients with heart involvement compared with patients of the other groups (*p* < 0.001), as shown in **Figure 6**. The AEC was also extremely high in patients with CNS involvement, but no statistical analysis was done due to the limited data (**Figures 6**, **7**).

### Therapies

Fifty-five out of 79 (69.7%) of the enrolled patients received systemic steroids, with a mean PDN equivalent dose of 17.5 mg/day. 13/79 (16.5%) were treated with methotrexate, 6/79 (7.5%) with hydroxyurea, 5 (6.3%) with cyclosporin A, 4 (5.1%) with azathioprine, 3 (3.8%) with IV Immunoglobulins, and 2 (2.5%) with imatinib. Nineteen patients received mepolizumab (24.1%) and 3 (3.8%) received rituximab (**Table 3**).

**Table 3:** Distribution of treatments in the enrolled cohort.

Therapy	Patients, *n* (%)
Prednisone	55 (69.7%)
Methotrexate	13 (16.5%)
Mepolizumab	19 (24.1%)
Hydroxyurea	6 (7.5%)
Ciclosporin	5 (6.3%)
Azathioprine	4 (5.1%)
Rituximab	3 (3.8%)
Intravenous immunoglobulins	3 (3.8%)
Imatinib	2 (2.5%)

### Diagnostic delay and clinical outcome

Our patients’ diagnosis time was 4 ± 1.8 months, starting from the first outpatient visit. Unfortunately, the time between symptom onset and the diagnosis was longer, estimated at 21.2 ± 8 months. No deaths were seen in our cohort, nor was end-stage organ damage.

## Discussion

Hypereosinophilia is a pretty common reason for allergy and clinical immunology consultations. In most cases, HE is isolated and self-limiting, thus not representing any real challenge for diagnostic and therapeutic purposes.

In our real-world experience, more than two-thirds of the initially evaluated patients were excluded from the study, primarily because of a not-sustained HE, which aligns with what happens in other real-life scenarios (23).

In patients with sustained HE, a comprehensive search for potential organ damage is of the utmost importance due to the extremely high rate of organ damage and the risk of end-stage organ damage in the case of a diagnostic delay. About 80% of the enrolled cohort showed at least two damaged organs.

In the last few years, the routine use of a two-tailed approach for HES diagnosis and organ damage detection has become increasingly accepted and implemented (1, 24). Still, no studies have focused on the exact timing of the application of this approach, which is the novelty of our study.

In our experience, the two-tailed approach should be implemented from the first outpatient visit, simultaneously testing for diagnosis and organ damage.

The routine use of the two-tailed approach in our clinical practice, as suggested by many recommendations (15, 16), induced us to search for any possible underlying cause of HE. This led to a very low rate of idiopathic HES, diagnosed only in 10% of our patients, a percentage far below that reported in the published real-world data.

?>In about a fourth of our HES patients, the HE and the organ damage were sustained by an infection or a drug reaction, thus being classified as R-HES. This observation underlines the importance of our approach to HE, considering that the patients had been referred to our center for incidental findings of hypereosinophilia and not due to the suspicion of an underlying disease.

Moreover, 20% of our patients, despite a whole assessment, were diagnosed with single-organ HES, and about 12% showed no organ damage at all (HEus). These data are noteworthy, as it is widely known that many single-organ HES, mainly with gastrointestinal involvement, could then be diagnosed with multisystemic HES (12). Regardless, none of our single-organ HES patients showed any disease progression, but all the patients initially classified as HEus eventually developed organ involvement and were reclassified as i-HES or l-HES, a finding that raises the possibility that HEus may not represent a truly distinct clinical entity but rather an early, pre-symptomatic phase of HES. This observation underscores the need for vigilant long-term monitoring of patients with HEus and may prompt a reconsideration of current clinical approaches to this condition.

The high prevalence of organ damage progression in patients with HEus raises the question of the importance of a comprehensive diagnostic approach. Likewise, it may also justify, both from an economic and ethical point of view towards the patient, the invasive diagnostic tests that should be required at diagnosis and follow-up.

Finally, in terms of organ damage, systematically searching for any tissue involvement, regardless of clinical presentation, has led to early recognition of HE-related cardiac damage in 12% of our patients. This percentage is much higher than that reported in the literature, estimated at around 5% in asymptomatic patients, with a significant increase up to 20% during the follow-up (7).

Based on this finding, we can postulate that the early use of the two-tailed approach in clinical practice allowed us to detect organ damage at a very early stage. As a consequence of that, the early treatment could be a reasonable explanation for the excellent outcome we observed in our patients, as no deaths nor end-stage organ damage were seen in our ten-year period follow-up. This is a remarkable observation, as in much recently published data, the mortality rate was far higher, estimated to be up to 75% in the case of a diagnostic delay (25).

Regarding diagnostic delay, the HES diagnosis in our cohort was carried out in about 4 months, which is far shorter than observed in the literature (10), probably thanks to the comprehensive two-tailed approach we used since the first outpatient visit. On the other hand, no differences were seen between the time of symptom onset and the time of the outpatients’ clinic referral. This could be linked to the low global attention paid to hypereosinophilia and its related symptoms in many clinical settings, as well as the barriers patients often face when getting to a center with HE expertise (26–28).

However, our study has some limitations. First, the selection of the patients could be a critical point. The patients referred to our center came from hematologists, gastroenterologists, and oncologists, so patients with clearly depicted clinical pictures were already excluded. Furthermore, the relatively small sample size, although one of the most extensive European case series, may be responsible for some limitations in terms of statistical analysis and comparison between groups.

Finally, the lack of a control group for comparing the efficacy of the two-tailed approach could represent a confounder. The two-tails approach was implemented in all the patients as a clinical practice and wasn’t initially designed as a prospective collection study. Therefore, we assume that further prospective studies on a larger scale, including a control group, are necessary to determine the possible implications and effectiveness of the two-tailed method on early diagnosis and disease follow-up.

In conclusion, HE is a common reason for allergists’ and clinical immunologists’ consultations, and the two-tailed approach, testing at the same time for diagnosis and organ damage, should be implemented from the first evaluation of patients with HE. This will aim at reducing diagnostic delay and the risk of mistakes in the classification of HES subtypes, thus allowing prompt and tailored treatment, as well as better outcomes.

## Data Availability

The raw data supporting the conclusions of this article will be made available by the authors, without undue reservation.

## Glossary

ABPA: Allergic Bronchopulmonary Aspergillosis
Af: *Aspergillus fumigatus*
ALPS: Autoimmune Lymphoproliferative Syndrome
ANCA: Antineutrophil Cytoplasmic Antibodies
ASA: Acetylsalicylic Acid
CBC: Complete Blood Count
CD: Cluster of Differentiation
CPK: Creatine Phosphokinase
COPD: Chronic Obstructive Pulmonary Disease
CRP: C-Reactive Protein
CRSwNP: Chronic Rhinosinusitis with Nasal Polyps
CT: Computed Tomography
CNS: Central Nervous System
DRESS: Drug Rash with Eosinophilia and Systemic Symptoms
DLCO: Diffusing Capacity for Carbon Monoxide
EGPA: Eosinophilic Granulomatosis with Polyangiitis
ENT: Ear, Nose, and Throat
ESR: Erythrocyte Sedimentation Rate
FGFR1: Fibroblast Growth Factor Receptor 1
FIP1L1–PDGFRA: Fusion gene FIP1L1–PDGFRA
GI: Gastrointestinal
HBV: Hepatitis B Virus
HCV: Hepatitis C Virus
HE: Hypereosinophilia
HEus: Hypereosinophilia of Undetermined Significance
HES: Hypereosinophilic Syndrome
HIV: Human Immunodeficiency Virus
HRCT: High-Resolution Computed Tomography
i-HES: Idiopathic Hypereosinophilic Syndrome
LDH: Lactate Dehydrogenase
l-HES: Lymphocytic Hypereosinophilic Syndrome
m-HES: Myeloid Hypereosinophilic Syndrome
MRI: Magnetic Resonance Imaging
NT-pro-BNP: N-terminal pro b-type Natriuretic Peptide
PCM1-JAK2: Fusion gene PCM1–Janus Kinase 2
PDGFRα: Platelet-Derived Growth Factor Receptor Alpha
PDGFRβ: Platelet-Derived Growth Factor Receptor Beta
PNS: Peripheral Nervous System
TCR: T-Cell Receptor
TMP/SMZ: Trimethoprim/Sulfamethoxazole
US: Ultrasound
X-ray: X-ray Chest
