# Supplementary material for: From hypereosinophilia to hypereosinophilic syndrome: real-world application of a two-tailed approach for HES diagnosis
Source: Front Immunol. 2026 Jan 6;16:1735131. doi: 10.3389/fimmu.2025.1735131 (PMC12815814; doi:10.3389/fimmu.2025.1735131)
Supplement: Supplementary file 2 [file Table1.docx]

| **HES** | | | **Patients**  **N (%)** | **Females**  **N (%)** | **Age at diagnosis**  **Yrs (range)** | **Most commonly reported symptoms (decreasing order)** | **EOS, cells/mcl**  **(mean ± SD)** | **Total IgE, KU/L**  **(mean ± SD)** | **Organ damage (decreasing order)** | **Treatment**  **(decreasing order)** |
| --- | --- | --- | --- | --- | --- | --- | --- | --- | --- | --- |
| Reactive-HES | | | 19/79 (24%) | 6/19 (31.6%) | 56.15 (27-73) |  | 3257.3 ± 2363.2 | 1536.3 ± 1408.6 |  |  |
|  | | |  |  |  |  |  |  |  |  |
|  | Asthma and CRSwNP | | 5/19 (26.3%) | 2/6 (33.3%) | 50.4 (29-71) | Uncontrolled asthma  Severe CRSwNP | 2180.0 ± 476.5 | 878.6 ± 762.4 | Lung  ENT tract | ICS/LABA  MPN  MEPO |
|  | Helminth infection | | 9/19 (47.4%) | 4/6 (66.7%) | 57.5 (43-71) | Diarrhoea  Pruritus  Urticaria | 3410.0 ± 2756.9 | 2106.8 ± 1016.2 | GI tract  Skin | Albendazole  Ivermectin |
|  | Drug reaction | | 4/19 (21.0%) | 0 | 47.4 (27-67) | Purpura  Eczema  Fever  Pruritus | 2968.0 ± 832.5 | 60.5 ± 35.0 | Skin  Liver | MPN |
|  | Malignancy | | 1/19 (5.3%) | 0 | 73 | Anorexia | 3160.0 | 156 | - | CHT |
| Myeloid-HES | | | 1/79 (12.6%) | 0 | 46 | Cough | 2450 | 76 | Bone marrow  Lung | Imatinib |
| Lymphocitic HES | | | 10/79 (12.7%) | 4/10 (40%) | 63.8 (46-83) | Papular eczema  Urticaria  Asthma and cough  Rhinits | 4619 ± 2591 | 945.7 ± 543.1 | Bone marrow  Skin  Lung | MPN  Hydroxyurea  Cyclosporin  MEPO  RTX |
| Overlap HES | | | 15/79 (19.0%) | 6/15 (40%) | 58.21 (47-79) | Asthma and CRSwNP  Purpura  Neuropathy  Glomerulonephritis  Myocarditis  Coronaritis | 12162.0 ± 9943 | 1297 ± 1067 | Lung  ENT  Peripheral Nervous System  Skin  Kidney  Heart | MPN  DMARDs (MTX, AZA, CyA)  MEPO  IVIG  RTX |
| Idiopathic HES | | | 8/79 (10.1%) | 5/8 (62.5%) | 65.37 (29-83) | Eczema, urticaria  Paresthesias  Diarrhoea, abdominal pain | 4968.4 ± 1433.2 | 324.6 ± 122.6 | GI tract  Skin  PNS | MPN  Hydroxyurea  MTX  Imatinib |
| Single-organ HES | | | 16/79 (20.2%) | 7/16 (43.7%) | 44.9 (17-71) |  |  |  |  |  |
|  | | EGID | 7/16 (43.8%) | 2/7 (28.6%) | 32.57 (17-71) | Diarrhoea  Abdominal pain  Dyspepsia  Anorexia | 4111.4 ± 1542.4 | 451.3 ± 122.7 | GI tract | Budesonide  Hydroxiurea  DMARDs (MTX, AZA, CyA) |
|  | | CEP | 8/16 (50%) | 5/7 (71.4%) | 59.57 (23-70) | Cough  Dyspnea | 4125 ± 2800.4 | 277.4 ± 199.5 | Lung | MPN |
|  | | EoF | 1/16 (6.3%) | 0 | 67 | Myalgia | 3200 | 158 | Muscle | MPN  MTX |
| Hypereosinophilia of unknown significance (HEus) | | | 10/79 (12.7%) | 4/10 (40%) | 54 (16-74) | None  Allergic Rhinitis | 3123 ± 1542.8 | 177.3 ± 97.5 | - | - |

Supplementary table 1 - Different aetiologies of HES, demographic data, clinical manifestations, treatments and laboratory values in the enrolled cohort.

AZA - Azathioprine; CHT - Chemotherapy; CRSwNP – Chronic Rhinosinusitis with Nasal Polys; CyA – cyclosporine A; ENT Tract – Ear, Nose, Throat tract; GI tract – Gastrointestinal tract; ICS/LABA – Inhaled Corticosteroids/Long Acting Beta2 Agonist; IVIG – Intravenous Immunoglobulins; MEPO - Mepolizumab; MPN - Methylprednisolone; MTX - Methotrexate; RTX - Rituximab;
